# Dissecting implicit food-related behaviors in Binge Eating Disorder and obesity: insights from a mobile approach-avoidance framework

**DOI:** 10.3389/fpsyg.2024.1435624

**Published:** 2024-10-09

**Authors:** Enrico Collantoni, Valentina Meregalli, Umberto Granziol, Angelo Di Vincenzo, Marco Rossato, Serena Giovannini, Elisa Capobianco, Hilmar Zech, Roberto Vettor, Angela Favaro

**Affiliations:** ^1^Department of Neuroscience, University of Padua, Padova, Italy; ^2^Padova Neuroscience Center, University of Padua, Padova, Italy; ^3^Department of Medicine, University of Padua, Padova, Italy; ^4^Department of General Psychology, University of Padua, Padova, Italy; ^5^Department of Psychiatry and Psychotherapy, Technische Universität Dresden, Dresden, Germany; ^6^Department of Child and Adolescent Psychiatry, Psychosomatics and Psychotherapy, University of Würzburg, Würzburg, Germany

**Keywords:** eating disorders, Binge Eating Disorder, obesity, approach-avoidance bias, impulsivity, emotional eating

## Abstract

**Introduction:**

Bulimic episodes experienced by patients with Binge Eating Disorder (BED) might be sustained by an enhanced behavioral propensity to approach food stimuli.

**Methods:**

To test this hypothesis, automatic approach avoidance tendencies toward high-calorie foods (HCF), low-calorie foods (LCF), and neutral objects were assessed in a group of 23 patients with BED, and their performance was compared to the one of 17 patients with obesity without BED and a group of 32 normal weight participants. All participants performed a mobile approach-avoidance task in which they were required to approach and avoid different stimuli by respectively pulling their phone toward themselves or pushing it away. Reaction times were analyzed.

**Results:**

Results showed a significant three-way interaction between group, type of movement and stimulus. *Post-hoc* analyses revealed that all the groups displayed an approach bias toward HCF. Patients with BED and healthy controls also displayed an approach bias toward LCF, a bias that was absent in obese individuals without BED. Moreover, patients with BED were faster in approaching food stimuli, both HCF and LCF, compared to healthy controls.

**Discussion:**

These behavioral tendencies are quite consistent with the real-life attitudes of both BED patients and patients with obesity and might contribute to the maintenance of unhealthy eating habits such as binging in patients with BED and high-calorie diets in patients with obesity.

## 1 Introduction

Recent research suggests that automatic cognitive processes may play an important role in disordered eating behaviors, differently influencing restrictive and binge-eating patterns (Fürtjes et al., 2020). However, to date, the current literature exploring these dynamics remains limited and inconsistent, mainly due to small and heterogeneous experimental samples and a lack of robust and ecologically valid behavioral protocols (Paslakis et al., 2021).

A clinical condition that has recently received some attention in this regard is Binge Eating Disorder (BED). BED is a psychiatric disorder characterized by recurrent episodes of binge eating, defined as eating, in a discrete period of time, large amounts of food, and a sense of lack of control over eating. Binge eating episodes are also associated with dysfunctional behaviors and/or negative feelings and they are not associated with the recurrent use of inappropriate compensatory behaviors (American Psychiatric Association, 2013). As patients with BED usually do not compensate for food intake, it results in high rates of obesity. However, from a psychological and behavioral perspective, patients with BED present unique characteristics compared to patients with obesity without BED (Klatzkin et al., 2015).

Numerous studies have shown that binge-eating behaviors in patients with BED are sustained by an intricate interplay of psychological and cognitive factors, calling for a more in-depth exploration of its underlying mechanisms (Giel et al., 2022). From a behavioral perspective, current research suggests that binge-eating behaviors are underpinned by difficulties in inhibitory control mechanisms, manifesting in rash impulsive behaviors, a pattern that has not been observed in patients with obesity who do not exhibit binge eating (Svaldi et al., 2014; Giel et al., 2017). In addition to a reduced inhibitory control, some researchers suggested that patients with BED might also show an enhanced behavioral propensity toward food stimuli, possibly related to a heightened response of the reward system (Balodis et al., 2015). To investigate this aspect, some studies focused on automatic approach-avoidance tendencies.

The most common task for the study of approach-avoidance tendencies is the Approach-Avoidance task (AAT; De Houwer et al., 2001; Rinck and Becker, 2007). This task requires participants to perform approach and avoidance movements toward different categories of stimuli. After analyzing their reaction times (RT), it can be inferred whether participants present an approach or avoidance bias toward a specific category of stimuli. If participants are faster in approaching than avoiding a particular group of stimuli, it indicates an approach bias. Conversely, if avoidance movements are faster than approach movements, it suggests an avoidance bias.

Studies employing the AAT in the context of BED have utilized a computerized version of the task, with rather inconclusive results to date. According to Paslakis et al. (2017), individuals with BED tend to avoid low-calorie food cues, while patients with obesity without BED showed a tendency to approach low-calorie food. Interestingly, healthy individuals also exhibited an avoidance bias away from low-calorie food cues, similar to the obesity-BED group. In a second study from the same group the authors explored the role of negative mood induction on approach-avoidance tendencies toward high-calorie and low-calorie food stimuli in both BED and obesity without BED conditions, compared to healthy controls. The results showed that inducing a negative mood state decreased implicit avoidance biases to food cues only in BED patients with concomitant obesity, but not in BED without obesity and normal-weight controls (Krehbiel et al., 2021). This constitutes the first direct evidence of the impact of emotional states on implicit behavioral tendencies in BED, highlighting the need to study such biases with protocols that ensure high ecological validity. Additionally, it highlights the importance of mechanisms that are related to emotional regulation in the treatment of this disorder. In line with the need for more ecologically valid observations of approach and avoidance biases than those provided by desktop-based protocols, in recent years, some efforts have been made to develop experimental protocols based on different technologies, such as smartphones and virtual reality (Schroeder et al., 2016; Collantoni et al., 2023), or that are able to incorporate more complex kinematic measurements than RT to evaluate behavioral tendencies (Meregalli et al., 2023). In recent years, some studies have tested and employed a mobile-app version of the AAT, which measures movement RT using the smartphone's accelerometer (Zech et al., 2020). Using this mobile app-based version of the AAT with food stimuli, Collantoni et al. (2023) pointed out the presence of a bias toward food in a large sample from the general population, which was associated with participants' BMI. More specifically, participants with a higher BMI were slower at avoiding high-calorie foods and approaching low-calorie foods than those with a lower BMI. An association between the behavioral bias toward food and BMI was also observed by Zech et al. (2023), who reported that normal-weight individuals tend to approach food stimuli slower after a meal. Conversely, participants with overweight or obesity presented an increase in approach tendencies after meals. The advantages offered by using the mobile version of the AAT are particularly evident in the possibility of more naturalistic approach and avoidance movements and in the ability to conduct recruitment outside of the laboratory setting, thus offering better ecological validity compared to the desktop-based version. However, to date, no studies have employed this tool in clinical populations.

In this study, we aim to evaluate behavioral tendencies toward high-calorie foods, low-calorie foods, and neutral stimuli in experimental samples of patients with BED, in patients with obesity and without BED, and in a healthy control group. The influence of individual factors (i.e., hunger, time elapsed since the last meal, wanting, liking, and fear of specific foods), clinical variables (i.e., BMI), and psychological scores (anxiety, depression, stress, and impulsivity) have also been assessed. In line with previous literature, we can hypothesize the presence of a behavioral bias toward food in each of the three groups. Additionally, we hypothesize that certain specific psychological factors, such as stress, impulsivity, and depression, will be associated with the food bias, specifically in the BED group. The lack of consistent preliminary data in the literature prevents the formulation of specific hypotheses about differences in approach and avoidance movements between the groups. However, clinical observation and the intrinsically impulsive nature of binge-eating behavior might suggest the presence of a greater approach bias for food, and in particular for high-calorie foods in patients with BED.

## 2 Methods

### 2.1 Participants

The sample included 72 participants: 23 patients with BED (both with and without obesity), 17 patients with obesity without BED, and 32 healthy controls (HC). Patients with BED were recruited from the Eating Disorder Center of the Hospital of Padova (Via Giustiniani, 2−35128, Padova), and they all met full criteria for BED according to the DSM-5 (American Psychiatric Association, 2013). Patients with obesity were recruited from the Center for the Study and Integrated Treatment for Obesity of the University Hospital of Padova (Via Giustiniani, 2−35128, Padova). For participants in the BED group, the diagnosis was confirmed by clinicians who are expert in eating disorders. For participants in the OB groups the presence of a diagnosis of BED or other eating disorders was excluded. The participants of the HC group constituted a subgroup of participants from our previous study conducted on the general population (Collantoni et al., 2023), who met specific inclusion and exclusion criteria and who were comparable to the experimental groups in terms of age and gender. HC participants were recruited online through advertisement and the design of the study (Collantoni et al., 2023) was the same as that presented here, thus making its sample sufficient for use as a health control group. It was approved by the ethical committee of the University of Padova, and all participants provided informed consent and engaged in the same protocol as described here. Participants received no compensation for their participation. Data collection for the current study was conducted between April and November 2023, while healthy controls were recruited from June to December 2021. Inclusion criteria for all participants were: (1) 18 years or older; (2) being fluent in Italian. Inclusion criterion specific for the BED group was the presence of a diagnosis of BED confirmed by an expert clinician. Additional inclusion criteria specific for the OB group were: (1) having a BMI higher than 30; and (2) not fulfilling the diagnostic criteria for BED or other eating disorders. Additional inclusion criteria for the HC participants were: (1) having a BMI comprised between 18.5 and 24.9; and (2) having a score lower than 2.8 on the global scale of the Eating Disorder Examination Questionnaire (EDE-Q; Mond et al., 2008).

Written informed consent was provided by all participants. The study was approved by the ethical committee of the University of Padova (reference number: 4149) and was conducted in accordance with the latest version of the Declaration of Helsinki.

### 2.2 Mobile AAT application

The mobile AAT app was programmed in Java using Android Studio (Zech et al., 2020). It could be downloaded from the University of Padova website (http://aatmobile.neuroscienze.unipd.it/) and installed on any Android smartphone. Participants were provided with the link for downloading the app on their own device and they could start the application at any moment. Once started the application, participants had to provide written informed consent and confirm to be over 18 years old. Then, they were asked to report the following demographic and clinical information: age, education level, work condition, height, weight, and pharmacological treatment. To control for the effect of hunger, participants also reported the time passed since their last meal (in minutes) and the perceived level of hunger (on a scale from 1 to 5). Following this initial assessment, participants completed the approach-avoidance task, which is described in more detail in the following section. At the end of the task, they rated liking, wanting, and fear toward each of the food stimuli observed during the task using a five-point likert scale.

Lastly, participants completed a series of self-reported questionnaires: (1) the EDE-Q (Fairburn and Beglin, 1994; Calugi et al., 2017); (2) the Depression Anxiety Stress Scale (DASS-21; Lovibond and Lovibond, 1996); and (3) the UPPS Impulsive Behavior Scale short version (D'Orta et al., 2015). Questionnaires are better described in Section 2.2.2.

#### 2.2.1 Approach-avoidance task

**Figure 1:**
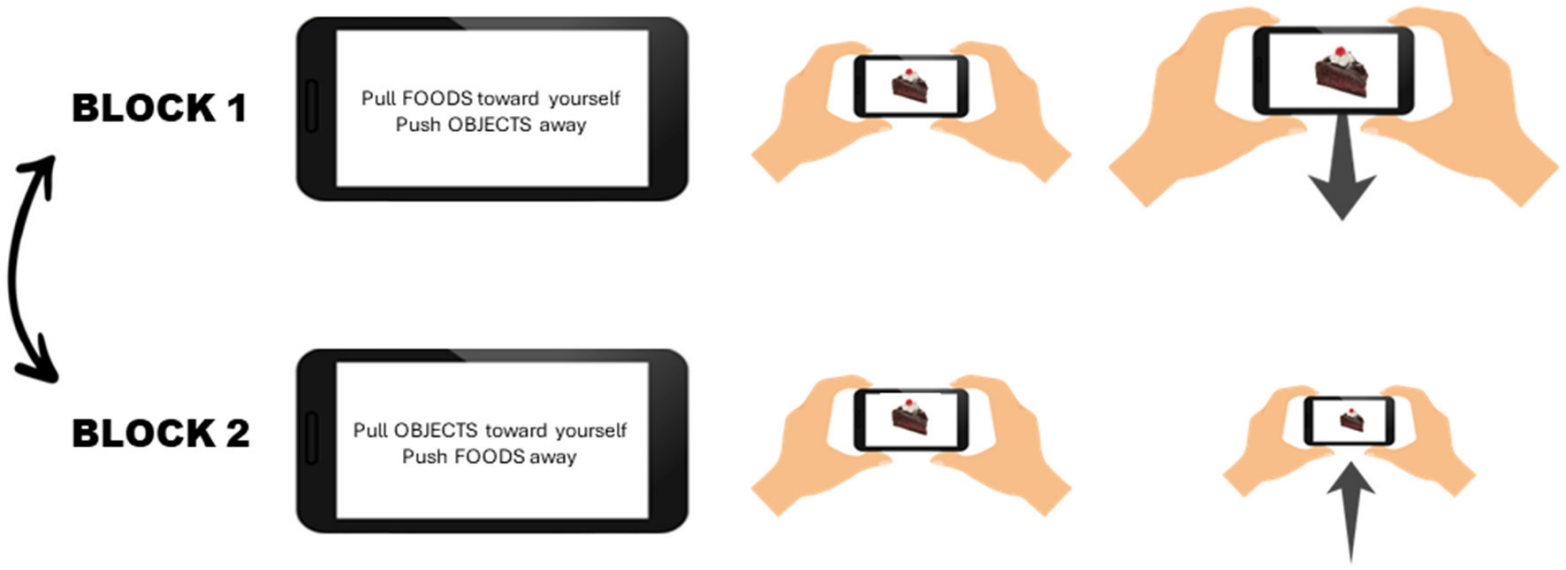
Experimental Setup. The task consists of two blocks, the order of which is randomized. In one block participants are instructed to pull food stimuli toward themselves and push objects away from themselves, while in the other block participants have to approach neutral objects and avoid food stimuli. During each block 20 pictures of each category (HCF, LCF, and neutral objects) are presented, for a total of 120 trials.

In the AAT, participants were required to approach or avoid specific stimuli by either pulling their phone toward themselves or pushing it away, as shown in Figure 1.

The stimuli comprised 15 pictures of high-calorie and high-processed foods (HCF), 15 pictures of low-calorie and low-processed foods (LCF), and 15 pictures of neutral objects (N). The pictures were all selected from the food.pics database (Blechert et al., 2019), and an analysis of their characteristics revealed that HCF pictures had a significantly higher intensity (*F* = 7.40, *p* = 0.002) and complexity (*F* = 10.89, *p* < 0.001) than LCF and neutral pictures.

Before starting the experiment, participants were provided with written instructions and two animated GIFs that displayed how to perform the approach and avoidance movements. The task was divided into two blocks. In one block, participants were instructed to pull food stimuli toward themselves and push objects away from themselves, while in the other block participants had to approach neutral objects and avoid food stimuli. The order of block presentation was counterbalanced between participants. During each block, 20 pictures of each category (HCF, LCF, and neutral objects) were presented. Pictures were selected randomly from our pool of images and could be repeated within and between blocks. Each block included 60 trials, for a total of 120 trials. At the beginning of each block, and in the middle of each block, participants were instructed as to which stimuli to approach and which ones to avoid, and they were asked to respond as fast as possible. Each trial started with a fixation point, displayed for 1,500 ms. Following the fixation point, a picture was displayed in the middle of the screen. If participants did not respond to the picture within 2 s, a clock was displayed on the screen to inform them that the trial had timed out. Before starting the real test, participants were provided with a series of additional practice trials, which were followed by a response feedback (an X for incorrect responses, and a V for correct responses). Participants could start the real test only after correctly responding to 16 practice trials.

For each trial, the phone's accelerometers and gyroscopes tracked the gravity- and rotation-corrected acceleration of the movement in the direction perpendicular to the face of the screen (100 Hz sampling rate). Based on the acceleration response, the accuracy and reaction time (RT) of each movement were calculated. The procedure to preprocess data was the same used by Zech et al. (2020).

#### 2.2.2 Self-reported questionnaires

*Eating disorder examination questionnaire*. The EDE-Q is a 28-item measure of eating disorder psychopathology. Each item is rated on a 7-point Likert scale from 0 to 6. Higher scores indicate greater severity. The scale generates four subscales: Restrain, Eating Concern, Shape Concern, and Weight Concern, and a global score. Scores ≥ 2.8 on the global EDE-Q score indicate probable clinical cases (Mond et al., 2008).

*Depression anxiety stress scale* (Lovibond and Lovibond, 1996). The DASS is a 21-item scale assessing symptoms of depression, anxiety, and stress. Each item is rated on a 4-point Likert scale from 0 to 3. Higher scores indicate greater severity. The scale generates three subscales: Depression, Anxiety, and Stress.

*UPPS impulsive behavior scale short version* (D'Orta et al., 2015). The UPPS is a 20-item scale assessing different facets of impulsivity. Each item is rated on a 4-point Likert scale, from 1 = strongly agree to 4 = strongly disagree. Higher scores indicate higher levels of impulsivity.

### 2.3 Statistical analysis

#### 2.3.1 Data exclusion

Following the procedure suggested by Zech et al. (2020), practice trials, error trials, trials with missing sensor data, and trials with RT below 200 ms or over two standard deviations from the mean RT were considered invalid. Participants with <80% valid experimental trials were excluded. In total, 11 participants were excluded.

#### 2.3.2 Data analysis

Data were analyzed through R statistical software, version 3.5.2. Statistical significance was determined using an alpha level of 0.05. To investigate the differences among the three groups (i.e., BED, OB, and HC) on the demographic and clinical data, Kruskal-Wallis sum rank tests were performed (Kruskal and Wallis, 1952), since the majority of these variables were not normally distributed. Wherever a test suggested a statistically significant effect, specific differences were tested through Dunn's tests (Dunn, 1964). For this last analysis, the FSA package was used (Ogle et al., 2023).

Furthermore, we investigated whether response times (RTs) for approach and avoidance movements varied depending on the type of stimulus presented. Due to the non-normal distribution of RTs, we opted to analyze our hypothesis using a generalized linear mixed-effect model (GLMM), specifically employing a Gamma distribution with an identity link function. Participant and Trial were treated as clustering and random variables. The use of mixed effect models was advantageous as they allowed for accounting for repeated measures and missing data. Deviation contrasts were applied for each fixed effect (group, movement type, and stimulus type), with the healthy control group and neutral food serving as reference levels, respectively. Additionally, we explored both two-way and three-way interactions among predictors. The GLMMs were implemented using the lme4 (Bates et al., 2015) package. To mitigate potential confounding effects, gender was incorporated into the model as a covariate. The formula for the model was: glmer [RT ~ stimulus type ^*^ movement type ^*^ group +gender+ (1 + stimulus type ^*^is_pull | participant) + (1|trial), family = Gamma (link=“identity”)]. To control for the effect of age, a follow-up analysis was performed adding also age as a covariate to the model. *Post-hoc* comparisons were conducted utilizing the emmeans package (Lenth, 2016). In this regard, to minimize the chance of committing Type 1 error, we tested only specific a-priori comparisons. Within the entire sample and within each group we tested (1) differences between approach and avoidance movements for each category of stimuli; (2) differences in RT for approach movements between the different stimuli; and (3) differences in RT for avoidance movements between the different stimuli. For the three-way interaction we also assessed differences between groups in RT of approach and avoidance movements for each category of stimuli.

Moreover, we tested if both category of food and group could have an effect also on the approach bias scores calculated as RT avoidance—RT approach. We also considered the interaction between predictors. In this case, we used a linear mixed effects model (LMM), setting participants as clustering variable. The model was tested through the lmerTest package (Kuznetsova et al., 2017).

Finally, we tested the correlations, divided by group, between bias indices (for HC foods, LC foods, and neutral objects) and the following variables: age, BMI, hunger, time since last meal, UPPS, total score of EDE-Q, and 3 subscales of DASS. We used spearman correlation coefficient. Given the number of correlations tested, we adjusted the *p*-values applying a Bonferroni correction method.

## 3 Results

### 3.1 Demographic and clinical characteristics

Five patients with BED, four patients with OB and two healthy controls were excluded due to too many invalid trials. The final sample included 18 patients with BED (*F* = 18), 13 patients with obesity without BED (*F* = 9), and 30 healthy controls (*F* = 23). Five patients with BED were taking antidepressants, three were taking benzodiazepine, and one was taking Risperidone. None of the healthy controls or patients with OB were taking any medication. Table 1 reports the demographic and clinical variables of the three groups. The analyses revealed that patients with obesity were significantly older compared to both BED patients and healthy controls. As regards BMI, no difference was observed between patients with BED and patients with obesity without BED. Patients with BED presented higher levels of anxiety, depression, and stress compared to healthy controls. Moreover, they also presented higher levels of impulsivity compared to both healthy controls and patients with obesity. Both patients with obesity and patients with BED presented higher levels of eating disorder psychopathology than healthy controls, as measured with the EDE-Q. No significant differences were observed between groups in hunger levels and time passed since last meal.

**Table 1:** Demographic and clinical data.

	**BED (18)**	**OB (13)**	**HC (30)**	**H**	***Post-hoc***
	**Mean (SD)**	**Mean (SD)**	**Mean (SD)**	**(** ***p*** **)**	
Sex (female)	18 (100%)	9 (69%)	23 (77%)		
Ethnicity					
Caucasian	17 (94%)	13 (100%)	30 (100%)		
African	1 (6%)				
Age (years)	27.22 (12.05)	49.62 (8.23)	27.33 (10.94)	**20.89 (<0.001)**	BED vs. OB (<0.001)
					OB vs. HC (<0.001)
BMI (kg/cm^2^)	32.14 (6.45)	41.65 (7.57)	21.51 (1.81)	**43.67 (<0.001)**	BED vs. HC (<0.001)
					OB vs. HC (<0.001)
DASS anxiety	12.88 (9.29)	6.92 (5.27)	4.76 (4.36)	**14.38 (<0.001)**	BED vs. HC (<0.001)
DASS depression	18.13 (11.04)	9.38 (7.18)	7.45 (7.35)	**11.80 (0.003)**	BED vs. HC (0.002)
DASS stress	19.00 (8.52)	12.77 (7.37)	12.55 (8.70)	**6.71 (0.035)**	BED vs. HC (0.038)
UPPS	51.31 (6.31)	38.62 (10.55)	43.87 (7.66)	**13.96 (<0.001)**	BED vs. HC (0.011)
					BED vs. OB (0.001)
EDE-Q total	3.82 (1.19)	2.44 (0.73)	0.93 (0.71)	**36.67 (<0.001)**	BED vs. HC (<0.001)
					OB vs. HC (0.003)
Time last meal (min)	154 (80.87)	264 (235.15)	144.42 (123.69)	2.29 (0.319)	
Hunger level	2.47 (1.28)	1.69 (0.85)	2.03 (1.10)	3.08 (0.214)	

BED, group of participants with Binge Eating Disorder (n = 18); OB, group of participants with obesity (BMI > 30) without BED (n = 13); HC, group of healthy controls without eating disorders (EDE-Q < 2.8) and with a weight in the normal range (BMI between 18.5 and 24.9).

BMI, Body Mass Index; DASS, Depression Anxiety Stress Scale; EDE-Q, Eating Disorder Examination-Questionnaire; UPPS, Impulsive Behavior Scale.

Statistical significance was determined using an alpha level of 0.05. Only significant *post-hoc* tests have been reported. Bold text indicates statistically significant results.

### 3.2 Approach avoidance task—Reaction times

**Figure 2:**
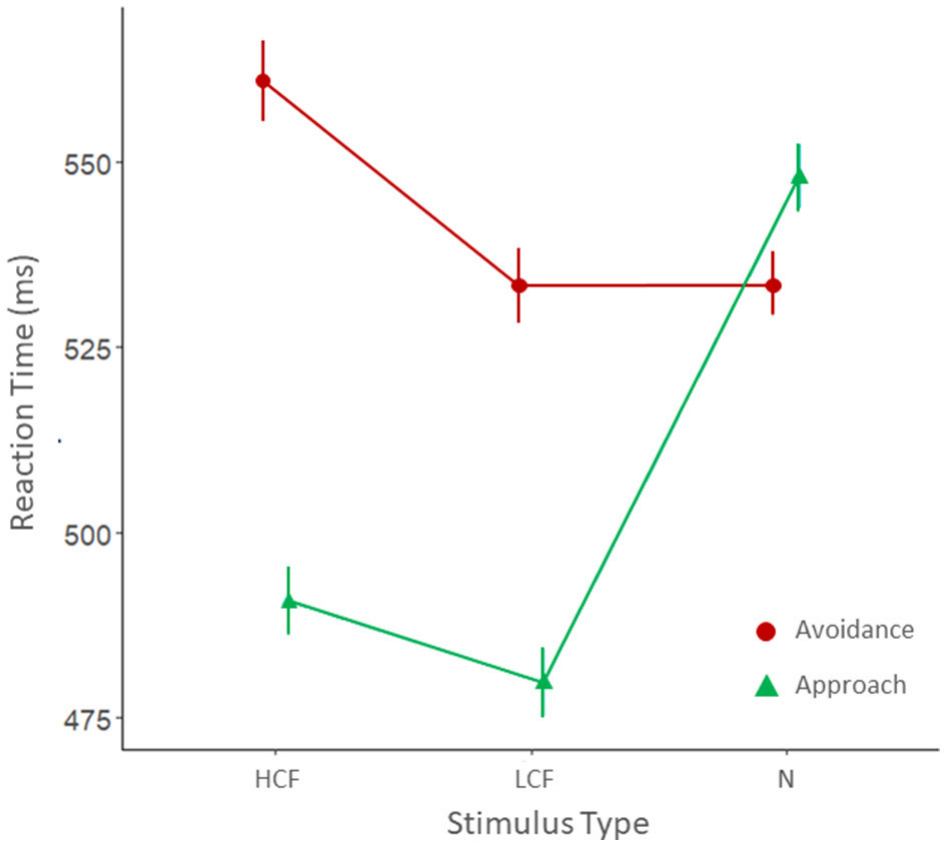
Mean reaction times for avoidance (red) and approach (green) movements for the three categories of stimuli: HCF, high-calorie foods; LCF, low-calorie foods; and N, neutral objects.

The results of the model revealed a significant two-way interaction between type of movement and stimulus (χ = 104.46, *p* < 0.001). In particular, as shown in Figure 2 and as revealed by the *post-hoc* analyses (Table 2) we can observe the presence of a general approach bias toward both HCF and LCF since participants were faster in approaching rather than avoiding these stimuli. On the contrary, this bias was not observed for neutral objects. Consistently, results showed that participants were faster in approaching both HCF and LCF than neutral objects. Lastly, *post-hoc* analyses revealed that participants were slower in avoiding HCF than LCF.

**Table 2:** *Post-hoc* contrasts for the type of movement by stimulus interaction.

	**Estimate (ms)**	***p***
HCF avd—HCF app	67.36	**<0.001**
LCF avd—LCF app	53.13	**<0.001**
N avd—N app	−7.49	1.000
HCF app—LCF app	10.45	1.000
HCF app—N app	−60.39	**<0.001**
LCF app—N app	−70.84	**<0.001**
HCF avd—LCF avd	24.68	**0.018**
HCF avd—N avd	14.47	0.849
LCF avd—N avd	−10.21	1.000

HCF, high-calorie foods; LCF, low-calorie foods; N, neutral objects; app, approach movement; avd, avoidance movement.

Statistical significance was determined using an alpha level of 0.05. Bold text indicates statistically significant results.

**Figure 3:**
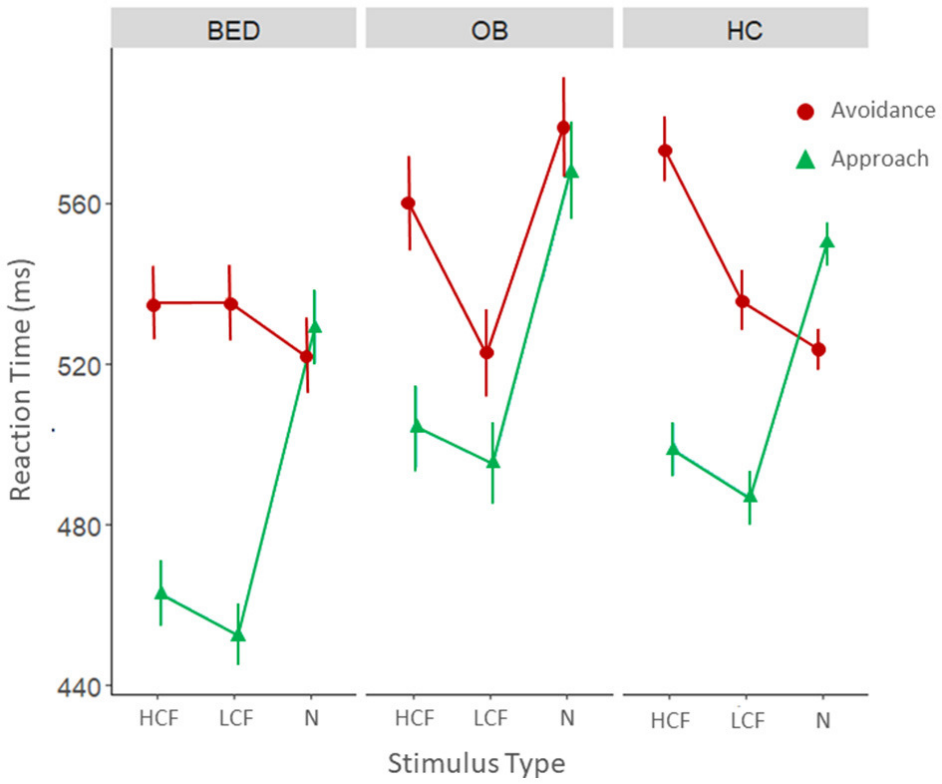
Mean reaction times for avoidance (red) and approach (green) movements for the three categories of stimuli divided by the three groups: BED, group of participants with Binge Eating Disorder (*n* = 18); OB, group of participants with obesity (BMI > 30) without BED (*n* = 13); HC, group of healthy controls without eating disorders (EDE-Q < 2.8) and with a weight in the normal range (BMI between 18.5 and 24.9).

The results of the model also showed that the 3-way interaction stimulus type X movement type X group was significant (χ = 10,217, *p* = 0.037; Figure 3).

As a first step we conducted a series of *post-hoc* analyses within the individual groups. As shown in Table 3 all the groups displayed an approach bias toward HCF. Patients with BED and healthy controls also displayed an approach bias toward LCF, while this bias was absent in patients with obesity. Unexpectedly, healthy controls also presented an avoidance bias toward neutral objects. Looking at approach movements, we can observe that participants of all the groups were faster in approaching food stimuli, both HCF and LCF, compared to neutral objects. Looking at avoidance movements we observed that participants with obesity were faster in avoiding LCF than neutral objects, while healthy controls were slower in avoiding HCF compared to both LCF and neutral objects.

**Table 3:** *Post-hoc* contrasts within the individual groups.

	**BED**	**OB**	**HC**
	**Estimate (ms) (** ***p*** **)**	**Estimate (ms)** ***(p)***	**Estimate (ms) (** ***p*** **)**
HCF avd—HCF app	**72.17 (<0.001)**	**55.54 (0.017)**	**74.39 (<0.001)**
LCF avd—LCF app	**82.69 (<0.001)**	27.50 (1.000)	**49.20 (<0.001)**
N avd—N app	−7.15 (1.000)	11.13 (1.000)	**−26.45 (0.012)**
HCF app—LCF app	10.28 (1.000)	8.97 (1.000)	12.11 (1.000)
HCF app—N app	**−66.10 (<0.001)**	**−63.73 (0.002)**	**−51.33 (<0.001)**
LCF app—N app	**−76.37 (<0.001)**	**−72.69 (<0.001)**	**−63.44 (<0.001)**
HCF avd—LCF avd	−0.25 (1.000)	37.01 (0.789)	**37.30 (0.017)**
HCF avd—N avd	13.22 (1.000)	−19.32 (1.000)	**49.51 (<0.001)**
LCF avd—N avd	13.47 (1.000)	**−56.32 (0.019)**	12.21 (1.000)

BED, group of participants with Binge Eating Disorder (n = 18); OB, group of participants with obesity (BMI > 30) without BED (n = 13); HC, group of healthy controls without eating disorders (EDE-Q < 2.8) and with a weight in the normal range (BMI between 18.5 and 24.9).

BMI, Body Mass Index; DASS, Depression Anxiety Stress Scale; EDE-Q, Eating Disorder Examination-Questionnaire; UPPS, Impulsive Behavior Scale; HCF, high-calorie foods; LCF, low-calorie foods; N, neutral objects; app, approach movement; avd, avoidance movement.

Statistical significance was determined using an alpha level of 0.05. Bold text indicates statistically significant results.

*Post-hoc* tests were also performed to assess differences between groups (Table 4). The results revealed that patients with BED were faster in approaching food stimuli, both HCF and LCF, compared to healthy controls, while we observed no significant differences in avoidance movements. Patients with BED were also faster in approaching LCF compared to participants with obesity. Lastly, results showed that patients with obesity were slower in avoiding neutral objects compared to both BED patients and controls. The follow up control analysis performed using the model with age as covariate confirmed the reported pattern of results.

**Table 4:** *Post-hoc* contrasts between groups.

	**BED vs. HC**	**BED vs. OB**	**OB vs. HC**
	**Estimate (ms) (** ***p*** **)**	**Estimate (ms)** ***(p)***	**Estimate (ms) (** ***p*** **)**
HCF avd	−38.04 (0.069)	−24.65 (1.000)	−13.38 (1.000)
LCF avd	−0.48 (1.000)	12.60 (1.000)	−13.09 (1.000)
N avd	−1.74 (1.000)	**−57.19 (0.006)**	**55.44 (<0.001)**
HCF app	**−35.81 (0.025)**	−41.28 (0.064)	5.47 (1.000)
LCF app	**−33.98 (0.031)**	**−42.59 (0.032)**	8.62 (1.000)
N app	−21.04 (1.000)	−38.91 (0.420)	17.87 (1.000)

BED, group of participants with Binge Eating Disorder (n = 18); OB, group of participants with obesity (BMI > 30) without BED (n = 13); HC, group of healthy controls without eating disorders (EDE-Q < 2.8) and with a weight in the normal range (BMI between 18.5 and 24.9).

BMI, Body Mass Index; DASS, Depression Anxiety Stress Scale; EDE-Q, Eating Disorder Examination-Questionnaire; UPPS, Impulsive Behavior Scale; HCF, high-calorie foods; LCF, low-calorie foods; N, neutral objects; app, approach movement; avd, avoidance movement.

Statistical significance was determined using an alpha level of 0.05. Bold text indicates statistically significant results.

### 3.3 Approach avoidance task—Bias score

**Figure 4:**
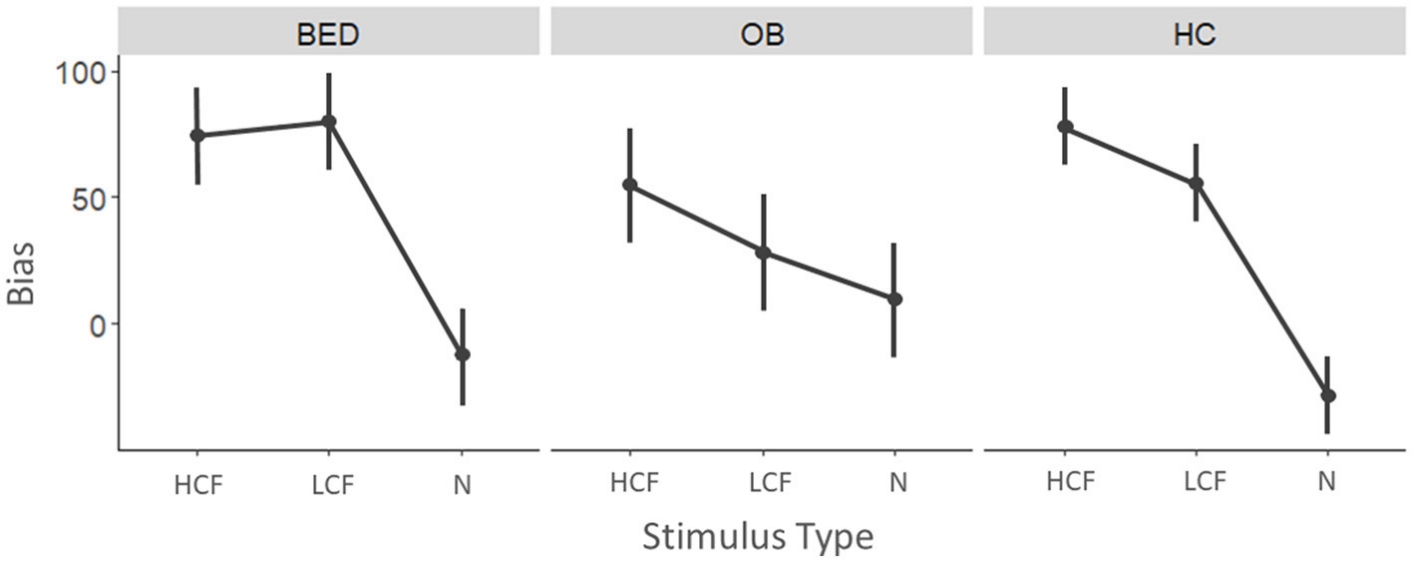
Mean approach bias scores for the three categories of stimuli divided by the three groups. Higher values indicate a stronger approach bias.

The model conducted on the approach bias score revealed no significant results (Figure 4).

As regards correlations, we observed that both patients with BED and healthy controls presented a positive correlation between time passed since last meal and bias toward LCF (BED: *r* = 0.591, *p* = 0.020; HC: *r* = 0.417, *p* = 0.034). Patients with BED also showed a significant positive correlation between the bias toward HCF and anxiety (*r* = 0.515, *p* = 0.041), stress (*r* = 0.678, *p* = 0.003), and impulsivity (*r* = 0.606, *p* = 0.013). However, only the correlation between bias toward HCF and stress survived correction for multiple comparisons (Supplementary Table 1).

## 4 Discussion

This study aimed at assessing automatic approach-avoidance tendencies elicited by both high-calorie and low-calorie food cues in three distinct groups: patients with BED, patients with obesity without BED and a group of healthy controls by means of a novel mobile-app based version of the AAT (Collantoni et al., 2023).

In line with previous literature, the analyses revealed a significant approach bias toward HCF cues in all three groups, likely confirming the saliency and the hedonic value of these stimuli, as participants are faster in approaching than in avoiding them (Gearhardt and DiFeliceantonio, 2023). A novel element that emerged from our study is that this bias was observed even for LCF stimuli, but only in patients with BED and HCs, while it was not observed in patients with obesity but without BED. This result, which differs from previous findings (Paslakis et al., 2017) suggests that individuals with obesity may present a specific motor preference for HCF, while low-calorie ones are processed without any differentiation in terms of implicit behavioral tendencies. Moreover, this effect is probably driven by a stronger implicit tendency to automatically avoid these stimuli, as our results indicate that individuals with obesity tend to avoid LCF cues faster than neutral ones. Overall, these data confirm a clinical-behavioral differentiation between BED and obesity that warrants further investigation (Bray et al., 2022). HC participants also displayed an avoidance bias for neutral objects. This result was unexpected, and it could be linked to non-specific factors that warrant further investigation.

An additional point of interest of the present research concerns the presence of specific behavioral patterns toward food stimuli in the three groups. In particular, healthy controls exhibit slower avoidance of HCF compared to neutral and LCF stimuli, likely due to less efficient processing of these high-calorie cues.

Moreover, participants with BED displayed specific behavioral patterns toward food in general, approaching it faster than healthy controls. The fact that these behavioral tendencies were not influenced by the calorie content of the stimuli suggests heightened impulsivity in individuals with BED as compared to those suffering from obesity. This aligns with previous literature indicating that impulsivity in BED is not specific to certain types of food but rather manifests in a more generalized manner (Giel et al., 2017) and underscores the need for a more through and detailed exploration of these behavioral patterns. Finally, our findings in the context of obesity seem to corroborate prior observations in patients with overweight, indicating the existence of approach and avoidance patterns that manifest toward food with specific caloric content and processing levels (Collantoni et al., 2023).

Interestingly, the implicit behavioral tendencies that emerged from this study are quite consistent with the real-life attitudes of both BED patients and patients with obesity and might contribute to the maintenance of unhealthy eating habits such as binge-eating behaviors in patients with BED and high-calorie diets in patients with obesity. This observation suggests the potential usefulness of implementing specific behavioral trainings aimed at modifying approach-avoidance tendencies toward foods. The efficacy of these approaches has already been suggested and documented in the literature, although further experimental evidence is needed (Cardi et al., 2022; Keeler et al., 2022). In this context, a modified version of our mobile task could be particularly useful, as it has been observed that mobile tasks are particularly effective in delivering intense and targeted trainings (Zech et al., 2022).

The correlation analyses with clinical, psychological, and demographic variables evidenced a potential influence of anxiety, stress, and impulsivity on the bias toward HCF cues in patients with BED. However, only the association with stress remains significant after adjusting for multiple comparisons. Although our findings are rather exploratory, they support existing evidence that psychological factors and emotional regulation might play a role in the physiopathology of BED.

The role of stress in the BED pathophysiology is still a topic of debate, with emerging results suggesting a potential involvement of stress-related factors in the neurobiology of the disorder (Naish et al., 2019). Other evidence suggests the possibility of stress playing a role in exacerbating binge-eating behaviors. Goldschmidt et al. (2014) for instance, using ecological momentary assessment, have highlighted that stress precedes the occurrence of binge-eating behaviors and that increases in negative affect following stressful events mediate this relationship. These findings underscore the importance of ecological assessments in understanding these mechanisms and suggest the need for future research that combines behavioral experimental paradigms with momentary appraisals. The implementation of tasks on mobile applications or on other portable technologies in this regard should be explored further.

This study has strengths and limitations, which must be considered in interpreting the data. The first limitation is related to the limited size of the experimental samples and the imbalance in numerosity between the patients and control groups, which makes the nature of this evaluation rather exploratory. A second limitation is due to the demographic heterogeneity of the samples; specifically, the patients with obesity included in the study are older than the patients in the other two groups, and thus they might present slower RT. Moreover, some of the patients with BED were taking medications that also might interfere with reaction times. However, since we were mainly interested in differences between approach and avoidance movements, overall RT should not impact the interpretation of our results. Another methodological limitation is due to the fact that the pictures depicting HCF were, on average, more intense and complex than LCF and neutral pictures. It is possible that these differences in visual characteristics may have affected how the participants processed the content of the pictures, which could have influenced their reaction times. However, since the main focus of the study was to examine the interactions between the type of stimulus (HCF, LCF, or neutral), the type of movement, and the participant group, any potential impact of the visual characteristics of the pictures on the conclusions of the study is likely to be minimal. Lastly, it should be acknowledged that this study measured response times to food pictures, not real foods. This reduces the ecological validity of the study as real foods also possess different sensory cues, such as smell. Moreover, it remains unclear whether these approach/avoidance behaviors translate to real-world food consumption. A strength of this research lies in using an experimental paradigm based on a mobile application, which ensures the possibility of more naturalistic approach and avoidance movements compared to desktop-based paradigms. Other strengths include using neutral stimuli alongside high and low-calorie foods and integrating various psychopathological, clinical-demographic, and intraindividual-based factors.

## 5 Conclusion

In conclusion, this study highlighted a significant approach bias toward HCF cues across all groups, with distinctive behavioral patterns observed in patients with BED and obesity. In particular, patients with BED tended to approach food stimuli faster than HCs, irrespective of their caloric value. The use of a mobile-app-based AAT provided insights into the role of calorie content in approach-avoidance behaviors and the influence of psychological factors like stress on eating disorders. Additional research is required to identify and describe behavioral biases within a broader ecological framework.

## Data Availability

The raw data supporting the conclusions of this article will be made available by the authors, without undue reservation.
