# Supplementary material for: Dissecting implicit food-related behaviors in Binge Eating Disorder and obesity: insights from a mobile approach-avoidance framework
Source: Front Psychol. 2024 Oct 9;15:1435624. doi: 10.3389/fpsyg.2024.1435624 (PMC11496131; doi:10.3389/fpsyg.2024.1435624)
Supplement: Supplementary file 1 [file Table_1.docx]

Supplementary Materials

| **Table S1.** Correlation between bias scores for HCF and LCF and clinical and demographic variables | | | | | | |
| --- | --- | --- | --- | --- | --- | --- |
|  | **BED-HCF** | **BED-LCF** | **OB-HCF** | **OB-LCF** | **HC-HCF** | **HC-LCF** |
| Age (years) | -.046 (.862) | -.195 (.453) | .146 (.635) | -.375 (.206) | -.088 (.644) | .068 (.721) |
| BMI (kg/cm^2^) | .051 (.844) | .181 (.487) | .132 (.667) | -.163 (.594) | -.087 (.649) | -.204 (.281) |
| Time last meal | .038 (.894) | **.591 (.020)** | -.019 (.951) | .064 (.834) | .288 (.154) | **.417 (.034)** |
| Hunger level | .301 (.241) | .426 (.089) | -.100 (.746) | -.032 (.918) | -.257 (.171) | -.122 (.519) |
| DASS anxiety | **.515 (.041)** | .165 (.541) | -.244 (.421) | .173 (.572) | -.116 (.550) | -.364 (.052) |
| DASS depression | .454 (.078) | .248 (.355) | .068 (.826) | .228 (.453) | -.109 (.572) | -.323 (.088) |
| DASS stress | **.677 (.004)^*^** | .240 (.370) | .196 (.523) | .436 (.136) | -.019 (.923) | -.035 (.856) |
| UPPS | **.606 (.013)** | .070 (.796) | .094 (.761) | -.144 (.640) | .088 (.644) | .023 (.903) |
| EDE-Q total | .089 (.744) | .042 (.877) | -.146 (.634) | -.150 (.624) | .089 (.649) | -.216 (.259) |
| BED, Binge Eating Disorder; OB, obesity; HC, healthy control; BMI, Body Mass Index; DASS, Depression Anxiety Stress Scale; EDE-Q, Eating Disorder Examination-Questionnaire; HCF, high calorie food; LCF, low calorie foods. *survived for multiple comparison correction. | | | | | | |

| **Table S2.** List of inclusion and exclusion criteria for the three groups | | |
| --- | --- | --- |
|  | **Inclusion Criteria** | **Exclusion Criteria** |
| BED | Age > 18 years  Fluent Italian  DSM 5 diagnosis of BED |  |
| OB | Age > 18 years  Fluent Italian  BMI > 30 | DSM-5 diagnosis of BED or ED |
| HC | Age > 18 years  Fluent Italian  18.5 < BMI < 24.5 | Score higher than 2.8 in the EDE-Q |
| BED, Binge Eating Disorder; OB, obesity; HC, healthy control; ED, eating disorder; BMI, Body Mass Index; EDE-Q, Eating Disorder Examination-Questionnaire. | | |
